# Antimicrobial Susceptibility and Virulence of *mcr-1*-Positive *Enterobacteriaceae* in China, a Multicenter Longitudinal Epidemiological Study

**DOI:** 10.3389/fmicb.2020.01611

**Published:** 2020-07-28

**Authors:** Bin Jiang, Pengcheng Du, Peiyao Jia, Enbo Liu, Timothy Kudinha, Hui Zhang, Dongxue Li, Yingchun Xu, Liangyi Xie, Qiwen Yang

**Affiliations:** ^1^Department of Clinical Laboratory, Peking Union Medical College Hospital, Peking Union Medical College, Chinese Academy of Medical Sciences, Beijing, China; ^2^Department of Clinical Laboratory, Hunan Provincial People’s Hospital, The First Affiliated Hospital of Hunan Normal University, Changsha, China; ^3^Beijing Key Laboratory of Emerging Infectious Diseases, Institute of Infectious Diseases, Beijing Ditan Hospital, Capital Medical University, Beijing, China; ^4^Graduate School, Peking Union Medical College, Chinese Academy of Medical Sciences, Beijing, China; ^5^School of Biomedical Sciences, Charles Sturt University, Orange, NSW, Australia

**Keywords:** *mcr-1*, intra-abdominal infections, urinary tract infections, resistance, virulence

## Abstract

This study was to investigate the prevalence of *mcr-1*-positive *Enterobacteriaceae* (MPE) in intra-abdominal infections (IAIs), urinary tract infections (UTIs), and lower respiratory tract infections (LRTIs) in China. A total of 6,401 *Enterobacteriaceae* isolates were collected consecutively from IAI, UTI, and LRTI patients in 19 hospitals across mainland China during 2014–2016. MPE isolates were screened by PCR detection for the *mcr* gene. The resistance profiles were tested by antimicrobial susceptibility test. All MPE isolates were characterized by pulsed-field gel electrophoresis (PFGE), multi-locus-sequence typing, O and H serotyping, and whole-genome sequencing. Among the 6,401 *Enterobacteriaceae* isolates, 17 *Escherichia coli* strains (0.27%) were positive for the *mcr-1* gene. The MPE prevalence rates in IAI, UTI, and LRTI patients were 0.34% (12/3502), 0.23% (5/2154), and 0% (0/745), respectively. The minimum inhibition concentrations (MICs) of colistin against 3 isolates were of 0.5–2 mg/L, and 4–8 mg/L against other 14 isolates. All the 17 isolates were susceptible to meropenem, imipenem, tigecycline, and ceftazidime/avibactam. The 17 MPE isolates belonged to 14 different ST types, and those that belonged to the same STs were not clonal by PFGE. The *mcr-1*-harboring plasmid of ten MPE isolates could transfer to the recipients by conjugation and the colistin MICs of the transconjugants ranged from 0.5 to 8 mg/L. M*cr-1*-carrying plasmids from the 17 MPE isolates could be grouped into four clusters, including 8 IncX4 type, 4 IncI2 type, 4 IncHI2A type, and 1 p0111 type. Multiple-drug resistance genes and virulence genes were detected. In conclusion, the prevalence of MPE in IAI, UTI, and LRTI were low in China, and no clonal transmission was identified in our study. Most MPE isolates exhibited low-level colistin resistance. However, our study indicated that MPE isolates always carried a variety of drug resistance and virulence genes, which should be paid more attention.

## Introduction

In the past few decades, multidrug-resistant (MDR) Gram-negative bacilli (mainly *Escherichia coli* and *Klebsiella pneumoniae*) increasingly caused infections and spread rapidly, which posed a major threat to global health (Mathers et al., 2015; Potter et al., 2016; Miao et al., 2018). Therefore, colistin is inevitably used in the clinic setting and has been regarded as the last-resort agent against MDR *Enterobacteriaceae* (Li et al., 2017). The *mcr-1* gene was firstly discovered in animals as early as 1980, but few researches have been performed to study its functions and characteristics (Shen et al., 2016). Liu et al. (2016) firstly reported that the *mcr-1* gene was related to the colistin resistance in *Enterobacteriaceae* isolates from animals and humans. They found that not only is the resistance mechanism of polymyxin caused by the chromosomal mutations but also the *mcr-1* gene is commonly carried by plasmids. The plasmid-harboring *mcr-1* could be horizontally transferred among *Enterobacteriaceae*. The *mcr-1*-positive *Enterobacteriaceae* (MPE) became a high threat to break the last line of defense against the MDR *Enterobacteriaceae*.

This novel finding caused widespread concern around the world (Liu et al., 2016). Subsequently, South America, Asia, Europe, Africa, and other countries have described *mcr-1* in *Enterobacteriaceae* strains isolated from animals, foods, water, humans, and other ecosystems (Li B. et al., 2018). The gene may be transmitted to human beings through direct contact via the food chain or between humans and animals (Chen et al., 2017). A report from Bolivia described a very high rate (38.3%) of *mcr-1* gene carriage in isolates from healthy children (7–11 years old) in rural areas with no sewage treatment systems and where the economy is dominated by subsistence agriculture and animal husbandry (Giani et al., 2018). This illustrates the wide spread of the *mcr-1* gene through food, animals, and the environment, to the population. It is understood that the *mcr-1* gene has been detected in humans, animals, food, and the environment in more than 30 countries on five continents (Soo et al., 2019). In addition, multiple subtypes of *mcr* genes, including *mcr*-1 to *mcr*-10, have been identified recently (Anyanwu et al., 2020; Wang et al., 2020). The prevalent subtypes in China were *mcr-1*, *mcr-2*, *mcr-3, mcr-4*, *mcr-5*, and *mcr-7* (Nang et al., 2019). Although multiple studies have been performed to investigate the prevalence of MPE in the breeding industry, few were conducted in hospital and human community.

In this study, we performed a multicenter study in China to describe the epidemiology of MPE isolates from patients of intra-abdominal infection (IAI), urinary tract infection (UTI), and lower respiratory tract infection (LRTI). We screened the *mcr* gene among over six thousand non-repetitive Enterobacteriaceae isolates from the three types of patients to uncover the prevalence and performed whole-genome sequencing to study the genomic characteristics of *mcr-1*-positive isolates.

## Materials and Methods

### Bacterial Isolates

We enrolled a total of 6,401 non-repetitive, consecutively collected *Enterobacteriaceae* isolates from patients with IAIs, UTIs, and LRTIs, in 19 tertiary hospitals in China from the years 2014 to 2016. The study was approved by the Human Research Ethics Committee of Peking Union Medical College Hospital (Number: S-K238). The majority of the intra-abdominal specimens were obtained during surgery, including paracentesis specimens and peritoneal fluid, as well as sample of gall bladder, appendix, abscesses, liver, and pancreas. UTI isolates were mainly obtained from culture of midstream urine, and LRTI isolates were cultured from qualified lower respiratory tract samples, such as sputum and bronchoalveolar lavage fluid. All duplicate isolates (same species from the same patient) were excluded. Isolates were first identified at the local hospitals by Vitek 2 and then shipped to the central clinical microbiology laboratory of the Peking Union Medical College Hospital for further identification using MALDI-TOF MS (Vitek MS, BioMe ìrieux, France).

### Multi-Locus Sequence Typing (MLST) and Pulsed-Field Gel Electrophoresis (PFGE)

Multi-locus sequence typing was conducted according to the protocol and primers on the MLST website^1^. PFGE was carried out by using XbaI. DNA fragments were electrophoresed by the CHEF-MAPPER XA PFGE system (Bio-Rad Laboratories, United States) for 19 h at 14°C, 6 V/cm, 120°, and switch from 2.16 to 63.8 s. PFGE images were analyzed by BioNumerics software (Version 7.6, Applied Maths, Belgium).

### O and H Serotypes

We used serum agglutination reaction to conduct O antiserum typing. The cooled bacterial suspension was mixed with each of the available O (O1-O180) antisera and observed for agglutination. A bacterial isolate that did not react with all O antisera was classified as non-typeable. H antiserum typing was carried out by PCR and sequencing. The *fliC* gene was amplified by PCR (primer sequence: *fliC*-F 5′-ATGGCACAAGTCATTAATACCCAAC-3′; *fliC*-R: 5′-CTAACCCTGCAGCAG AGACA-3′). The sequences of PCR products were compared with Serotype Finder public database^2^.

### Antimicrobial Susceptibility Test

The antimicrobial susceptibility testing included nine antibiotics (colistin, meropenem, ertapenem, amikacin, levofloxacin, ceftazidime, tigecycline, aztreonam, ceftazidime/avibactam). The MICs of *Enterobacteriaceae* isolates in this study were determined by standard broth microdilution method. The results were interpreted according to Clinical and Laboratory Standards Institute (CLSI) standard M100-S29 (CLSI, 2019), except for colistin which was interpreted by European Committee on Antimicrobial Susceptibility Testing (EUCAST) breakpoints (EUCAST, 2016) and tigecycline by FDA-approved breakpoints. The quality control strains were *E. coli* ATCC25922 and *Pseudomonas aeruginosa* ATCC27853.

### Screening of the Isolates for *mcr-1* and Other Antimicrobial Resistance Genes

All strains were screened for the presence of the *mcr-1* gene by PCR as described previously (Liu et al., 2016). The resulting amplicons were verified by Sanger sequencing. We also screened all MPE isolates for the presence of ESBL genes (*bla*_*CTX–M*_, *bla*_*SHV*_, *bla*_*TEM*_), ampC (*bla*_*MOX*_, *bla*_*CMY*_, *bla*_*LAT*_, *bla*_*BIL*_, *bla*_*DHA*_, *bla*_*ACC*_, *bla*_*MIR*_, *bla*_*FOX*_, *bla*_*ACT*_) and carbapenemase genes (*bla*_*IMP*_, *bla*_*VIM*_, *bla*_*NDM*_, b*la*_*AIM*_, *bla*_*SPM*_, *bla*_*KPC*_, *bla*_*DIM*_, *bla*_*BIC*_, *bla*_*GIM*_, *bla*_*SIM*_, *bla*_*OXA–48*_) by PCR using primer and reaction conditions as previously described (Pérez-Pérez and Hanson, 2002; Queenan and Bush, 2007; Poirel et al., 2011; Hamprecht et al., 2016). The obtained gene sequences were compared with those in the nt database, using the NCBI blast server^3^.

### Conjugation Experiments

Conjugation experiments were carried out to investigate the transferability of the *mcr-1* gene in the MPE isolates. The 17 MPE isolates were used as the donors, and the rifampicin-resistant *E. coli* C600 strain was used as the recipient. The donor and recipient bacteria were incubated to TSB broth and cultured at 35°C for 10–12 h together. The transconjugants were screened on containing colistin (2 μg/ml) and rifampicin (600 μg/ml).

### Whole-Genome Sequencing of MPE Isolates and Complete Sequencing of the *mcr-1*-Carrying Plasmids

The genomic DNA of the MPE isolates was extracted using the Qiagen DNA Mini Kit (Qiagen, Hilden, Germany) and sequenced on the Illumina HiSeq 2500 platform. The genomes were assembled using SPAdes v3.13 by the default parameters (Bankevich et al., 2012). The complete genome sequence of *E. coli* strain EDL933 was used as the reference to perform phylogenetic analysis. The single-nucleotide polymorphisms (SNPs) were identified using MUMmer (Delcher et al., 2003). The adjacent mutations within 5-bp in an isolate were filtered to avoid recombination. Finally, the concatenated sequences of variant sites (core genome SNPs) conserved in all strains were used to perform phylogenetic analysis using FastTree (Price et al., 2009). The sequences were also determined by the Oxford Nanopore Technologies (ONT) MinION platform and then combined with the Illumina HiSeq 2500 platform-determined sequence. The Illumina read and ONT read were assembled with a hybrid strategy using Unicycler (Wick et al., 2017). Bioinformatic tools were used to analyze these genomes, including ResFinder for resistance genes (Kleinheinz et al., 2014), ISfinder for ISs (Siguier et al., 2006), and PlasmidFinder for plasmid replicon types (Carattoli et al., 2014). Sequence comparisons were performed using BLAST software. The sequences of the plasmids were annotated with Prokka (Seemann, 2014) and edited manually.

### Virulence Factor (VF) Determination

The presence of 29 virulence genes associated with extraintestinal pathogenic *E. coli* was detected by multiplex PCR as described previously (Johnson and Stell, 2000). The tested virulence genes included adhesin VF genes (*fimH*, *papG* I, *papG* II, *III*, *papG* allele I, *papG* allele II, *papG* allele III, *papAH*, *papC*, *papEF*, *sfaS*, *focG*, *bmaE*, *gafD*, *nfaE*, *sfa/focDE*, *afa*/*draBC*), toxin genes (*hlyA*, *cnf1*, *cdtB*), siderophore genes (*fyuA*, *iutA*), capsule synthesis-associated VF genes (*kpsMT* II, *kpsMT* III, *kpsMT* K1, *kpsMT* K5), and miscellaneous VF genes (*rfc*, *ibeA*, *cvaC*, *traT*, PAI). Pathogenicity island (PAI) from strain CFT073 was used as a PAI marker.

## Results

### Bacterial Isolates

In total, we collected 3,502 *Enterobacteriaceae* isolates from IAI patients, 2,154 from UTI patients, and 745 from LRTI patients. The total 6,401 non-repetitive isolates included the following species: *E. coli* (3598), *K. pneumoniae* (1840), *Enterobacter cloacae* (359), *Proteus mirabilis* (162), *Enterobacter aerogenes* (124), *Klebsiella oxytoca* (106), *Citrobacter freundii* (103), *Serratia marcescens* (100), and *Proteus vulgaris* (9). By PCR screening of the *mcr-1* gene, we found that 17 isolates (17/6,401, 0.27%) were positive for *mcr-1*, all of which were *E. coli*, accounting for 0.47% in this species (17/3,598). Among the 17 MPE isolates, 12 were from IAI patients, 5 from UTI patients, and none from LRTI patients, accounting for the prevalence rates of 0.34% (12/3501), 0.23% (2/2154), and 0% (0/745) among the three types of infection, respectively.

### Subtyping of the MPE Isolates and Whole-Genome Sequencing

**FIGURE 1:**
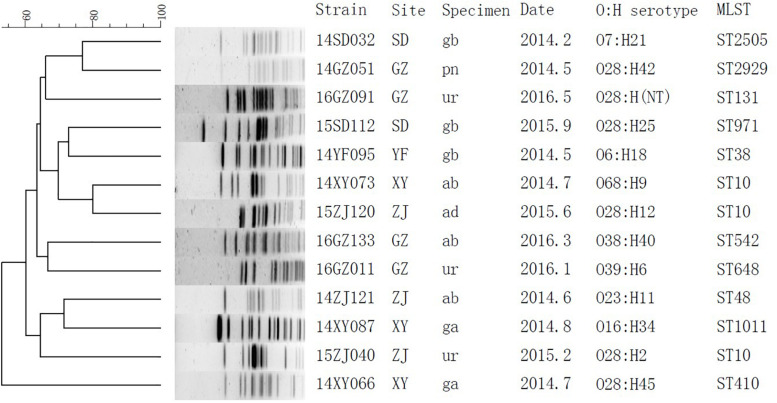
Phylogenetic tree of *mcr-1*-positive *Enterobacteriaceae* by PFGE analysis. SD, Shandong; GZ, Guangzhou; YF, Zhejiang; XY, Changsha; ZJ, Zhejiang; gb, gallbladder; pn, pancreas; ur, urine; ab, abdominal fluid; ad, abscess, abdominal; ga, gastric fluid.

The subtypes of the MPE isolates were analyzed by the PFGE, MLST, and O, H typing. According to the MLST analysis, 17 MPE isolates were grouped into 14 distinct sequence types (STs). Three strains belonged to ST10, two strains belonged to ST393, and the other 12 strains belonged to different types. In the PFGE test, 4 of the 17 MPE strains had no complete bands despite several repeats, and the other 13 strains were divided into 13 different PFGE types (Figure 1). The O and H serotype of 17 isolates included 14 different types and 3 untypable strains.

By whole-genome sequencing, we obtained 1.77 ± 0.17 Gb Illumina read data for the MPE isolates. We included 329 published genomes of *mcr-1*-positive *E. coli* and obtained 525,318 core genome SNPs by the SNP analysis. By the phylogenetic analysis (Supplementary Figure S1), the MPE isolates obtained in this study were clustered with the genomes of the same STs. The SNP numbers between ST393 strain 14ZS127 and 14TJ040 were 340, and those among ST10 strains were 1376 between 15ZJ120 and 15ZJ040, 2872 between 15ZJ120 and 14XY073, and 2914 between 15ZJ040 and 14XY073. Other 12 MPE strains all belonged to other 12 different STs and spread dispersedly in the tree. The results revealed no clonal transmission in the MPE isolates we obtained.

### Antimicrobial Susceptibility Test and Resistance Genes Detection

Among the 17 MPE isolates, 14 strains display low-level resistance to colistin (MIC: 4–8 mg/L) and the other 3 strains showed colistin MIC of 0.5–2 mg/L. All MPE isolates were susceptible to meropenem, imipenem, tigecycline, and ceftazidime/avibactam, and only one isolate was non-susceptible to amikacin. Six isolates (35.3%) were resistant to ceftazidime. Seven isolates (41.1%) were resistant to aztreonam. Most of the MPE isolates (82.4%) were resistant to levofloxacin. The detection of resistance genes by PCR revealed that all of the 17 MPE isolates carried multiple antimicrobial resistance genes, including *bla*_*TEM–1B*_ (13/17, 76.5%), *bla*_*CTX–M–14*_ (5/17, 29.4%), *bla*_*CTX–M–55*_ (4/17, 23.5%), *bla*_*TEM–209*_ (2/17, 11.8%), *bla*_*OXA–1*_ (2/17, 11.8%), *bla*_*CTX–M–132*_ (1/17, 5.9%), *bla*_*CTX–M–65*_ (1/17, 5.9%), *bla*_*TEM–135*_ (1/17, 5.9%), *bla*_*CTX–M–27*_ (1/17, 5.9%), and *bla*_*CMY–2*_ (1/17, 5.9%) (Table 1).

**TABLE 1:** Clinical and genetic characteristics of 17 *mcr-1*-positive *Enterobacteriaceae.*

**Isolate**	**Characterization**						
	**City**	**Specimen type**	**Date of isolation**	**Sequence type**	**mcr and beta-lactamase genes**	**O and H serotype**	**Inc type of mcr-1 carrying plasmid**
14ZJ112	Zhejiang	Gastric fluid	2014.5	ST46	*mcr-1*, *bla*_TEM–__1__B_, *bla*_TEM–__209_, *bla*_CTX–M–__55_	O_114_:H_(NT)_^2^	IncX4
14ZJ121	Zhejiang	Abdominal fluid	2014.6	ST48	*mcr-1*, *bla*_CTX–M–__14_	O_23_:H_11_	IncHI2A
14ZJ121-T^1^	NA	NA	NA	NA	*mcr-1*	NA	IncHI2A
14XY066	Changsha	Gastric fluid	2014.7	ST410	*mcr-1*, *bla*_TEM–__1__B_	O_28_:H_45_	IncX4
14XY066-T	NA	NA	NA	NA	*mcr-1*	NA	IncX4
14XY073	Changsha	Abdominal fluid	2014.7	ST10	*mcr-1*	O_68_:H_9_	IncX4
14XY073-T	NA	NA	NA	NA	*mcr-1*	NA	IncX4
14TJ040	Tianjin	Urine	2014.5	ST393	*mcr-1, bla*_TEM–__1__B_, *bla*_CTX–M–__132_	O_28_:H_1_	IncI2
14TJ040-T	NA	NA	NA	NA	*mcr-1*, *bla*_CTX–M–__132_	NA	IncI2
14XY087	Changsha	Gastric fluid	2014.8	ST1011	*mcr-1*, *bla*_TEM–__135_	O_16_:H_34_	IncI2
14XY087-T	NA	NA	NA	NA	*mcr-1*	NA	IncI2
14ZS127	Zhongshan	Urine	2014.4	ST393	*mcr-1*, *bla*_TEM–__1__B_, *bla*_CTX–M–__55_	O_116_:H_1_	IncX4
14ZS127-T	NA	NA	NA	NA	*mcr-1*, *bla*_CTX–M–__55_	NA	IncX4
14GZ051	Guangzhou	Pancreas	2014.5	ST2929	*mcr-1*, *bla*_CTX–M–__14_	O_28_:H_42_	IncHI2A
14GZ051-T	NA	NA	NA	NA	*mcr-1*, *bla*_CTX–M–__14_	NA	IncHI2A
14YF095	Zhejiang	Gallbladder	2014.5	ST38	*mcr-1*, *bla*_TEM–__1__B_, *bla*_CTX–M–__14_	O_6_:H_18_	IncX4
14YF095-T	NA	NA	NA	NA	*mcr-1*, *bla*_CTX–M–__14_	NA	IncX4
14SD032	shandong	Gallbladder	2014.2	ST2505	*mcr-1*, *bla*_TEM–__1__B_, *bla*_TEM–__209_, *bla*_CTX–M–__55_	O_7__:_H_21_	IncI2
15ZJ120	Zhejiang	Abscess, abdominal	2015.6	ST10	*mcr-1*, *bla*_TEM–__1__B_, *bla*_OXA–__1_, *bla*_CTX–M–__27_, *bla*_CMY–__2_	O_28_:H_12_	p0111
15ZJ040	Zhejiang	Urine	2015.2	ST10	*mcr-1*, *bla*_TEM–__1__B_, *bla*_OXA–__1_, *bla*_CTX–M–__65_	O_28_:H_2_	IncX4
15SD112	Shandong	Gallbladder	2015.9	ST971	*mcr-1*, *bla*_TEM–__1__B_	O_28_:H_25_	IncX4
16JL150	Jilin	Abscess	2016.2	ST156	*mcr-1*, *bla*_TEM–__1__B_	O_(NT)_:H_28_	IncHI2A
16JL150-T	NA	NA	NA	NA	mcr-1	NA	IncHI2A
16GZ133	Guangzhou	Abdominal fluid	2016.3	ST542	*mcr-1*, *bla*_TEM–__1__B_, *bla*_CTX–M–__14_	O_38_:H_40_	IncX4
16GZ091	Guangzhou	Urine	2016.5	ST131	*mcr-1*, *bla*_TEM–__1__B_, *bla*_CTX–M–__14_	O_28_:H_(NT)_	IncHI2A
16GZ091-T	NA	NA	NA	NA	*mcr-1*, *bla*_CTX–M–__14_	NA	IncHI2A
16GZ011	Guangzhou	Urine	2016.1	ST648	*mcr-1*, *bla*_TEM–__1__B_, *bla*_CTX–M–__55_	O_39_:H_6_	IncI2

*T: transconjugant; NT: non-typeable.*

### The *mcr-1*-Carrying Plasmids and Their Mobility

**FIGURE 2:**
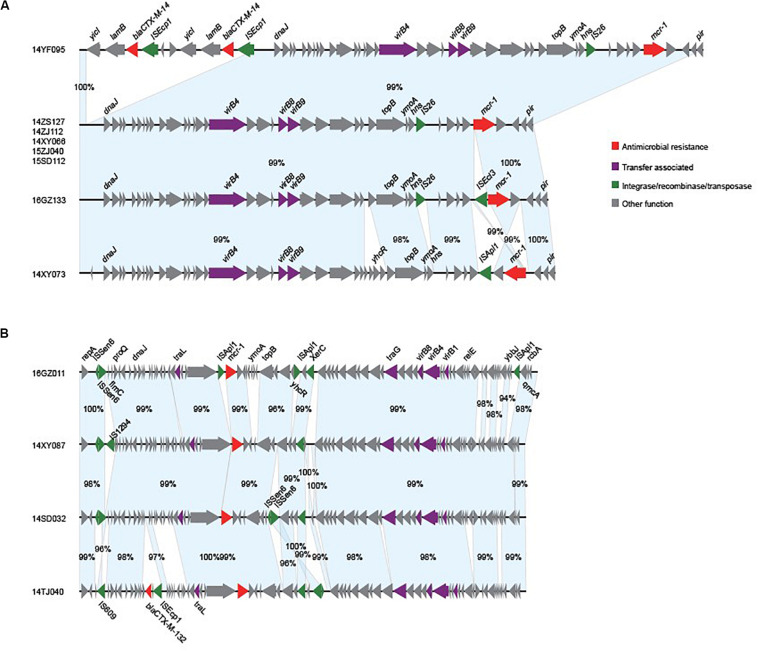
Sequence annotation and alignment of the *mcr-1*-carrying IncX4 plasmids **(A)** and IncI2 plasmids **(B)** obtained in this study. The arrows and triangles represent genes of different functional categories (red: antimicrobial resistance; green: integrase, recombinase, and transposase; purple: transfer associated; gray: other functions). The alignments between two plasmids are represented by light blue blocks, and the sequence identities are marked on the blocks.

**FIGURE 3:**
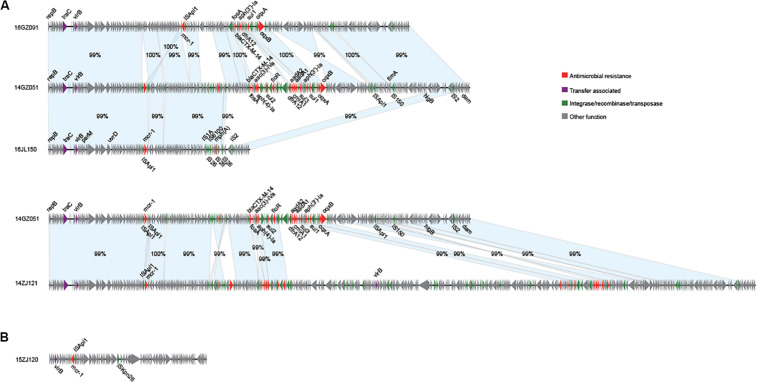
Sequence annotation and alignment of the *mcr-1*-carrying IncHI2A plasmids **(A)** and p0111 type plasmid **(B)** obtained in this study. The arrows and triangles represent genes of different functional categories (red: antimicrobial resistance; green: integrase, recombinase, and transposase; purple: transfer associated; gray: other functions). The alignments between two plasmids are represented by light blue blocks, and the sequence identities are marked on the blocks.

We obtained the complete sequences of the 17 *mcr-1*-carrying plasmids from the 17 MPE isolates. Based on the types of origin of replication and sequence similarity of the plasmids, these plasmids could be grouped into four clusters (Figures 2, 3). Eight plasmids belonged to the IncX4 type. Seven of the IncX4 plasmids were highly similar, of which the sizes were 33–34 kb (Figure 2A). In p14YF095-mcr, a 12-kb insertion fragment encoding two copies of *bla*_*CTX–M–14*_-IS*Ecp1* elements were identified. Four plasmids belonged to the IncI2 type, of which the sizes were 65–67 kb (Figure 2B). Multiple types of origin of replication were identified among other four plasmids, p14GZ051-mcr, p14ZJ121-mcr, p16JL150-mcr, and p16GZ091-mcr, and IncHI2A was the common type of origin of replication in the four plasmids (Figure 3A). The plasmid p15ZJ120-mcr belonged to the p0111 type (Figure 3B).

The *mcr-1* genes in eight plasmids were identified to be adjacent to IS elements. ISApl1 was the most common element identified in seven plasmids, including an IncX4 plasmid (p14XY073-mcr), an IncI2 plasmid (p16GZ011-mcr), all of the four IncHI2A plasmids, and the p0111 plasmid p15ZJ120-mcr. In plasmid p16GZ133-mcr, the *mcr-1* gene was adjacent to IS*Ecl3*. In other plasmids, no IS element was identified adjacent to *mcr-1*.

The *mcr-1* genes of 10 MPE strains were successfully transferred to recipient strain *E. coli* EC600. The transfer frequencies of the 10 mcr-1-harboring plasmids varied from 10^–2^ to 10^–4^. The colistin MIC range of the transconjugants was 0.5–8 mg/L (Table 2).

**TABLE 2:** MICs of 17 *mcr-1*-positive *Enterobacteriacea* and 10 transconjugates.

**Isolates**	**MIC (μg/ml)**								
	**COL**	**IPM**	**AZT**	**CAZ**	**T/A**	**AMK**	**LVX**	**MEM**	**TGC**
14ZJ112	4	0.25	0.06	0.25	0.12	16	>8	0.03	0.5
14ZJ121	2	0.12	2	0.5	≤0.015	2	0.5	0.03	0.25
14ZJ121-T	2	0.25	0.12	0.5	0.25	1	0.5	0.03	0.03
14XY066	4	0.12	0.12	0.25	0.03	4	>8	0.03	0.25
14XY066-T	4	0.25	0.25	0.25	0.25	1	0.5	0.03	0.12
14XY073	4	0.25	0.06	0.12	0.06	8	4	0.03	0.25
14XY073-T	4	0.25	0.12	0.5	0.25	1	0.5	0.03	0.12
14TJ040	8	0.25	128	128	0.06	8	>8	0.03	0.12
14TJ040-T	8	0.12	64	32	0.25	2	>8	0.03	0.12
14XY087	4	0.25	0.12	0.25	0.06	4	>8	0.03	1
14XY087-T	8	0.12	0.25	0.5	0.5	1	0.25	0.03	0.12
14ZS127	8	0.25	128	64	0.03	8	>8	0.03	0.12
14ZS127-T	8	0.25	64	32	0.12	2	>8	0.06	0.12
14GZ051	4	0.12	4	1	0.06	8	>8	0.015	0.25
14GZ051-T	4	0.25	4	1	0.25	2	1	0.06	0.06
14YF095	4	1	128	64	1	2	>8	0.12	2
14YF095-T	4	0.25	64	16	0.25	1	0.25	0.03	0.06
14SD32	4	0.25	64	16	0.03	>32	>8	0.03	0.25
15ZJ120	8	0.5	32	16	0.12	8	>8	0.03	0.5
15ZJ040	8	0.25	16	2	0.12	4	>8	0.03	1
15SD112	4	0.12	0.03	0.25	0.06	4	0.5	0.03	0.5
16JL150	4	0.06	0.12	0.5	0.06	2	>8	0.015	2
16JL150-T	0.5	0.25	0.12	0.5	0.25	1	0.5	0.03	0.03
16GZ133	0.5	0.12	8	2	0.12	4	0.03	0.03	0.5
16GZ091	8	0.12	4	1	0.06	4	4	0.03	0.5
16GZ091-T	4	0.25	2	0.5	0.25	0.5	>8	0.03	0.25
16GZ011	0.5	0.12	>128	>128	≤0.015	2	>8	0.5	0.5

*COL: colistin, IPM:imipenem, AZT: Aztreonam, CAZ: Ceftazidime, MEM: meropenem, TGC: tigecycline, AMK: amikacin, LVX: Levofloxacin T/A: ceftazidime/avibactam.*

### Virulence Gene Detection

Seventeen MPE isolates carried diverse virulence genes. The most prevalent gene was *fimH* (16/17, 94.1%), followed by *papA* (11/17, 64.7%), *kpsMT* III (10/17, 58.8%), *papG* allele III (8/17,47.7%), *traT* (7/17, 41.2%), *papEF* (6/17, 35.3%), *kpsMT* K1 (5/17, 29.4%), *kpsMT* II (4/17, 23.5%), and *bmaE* (4/17, 23.5%). The remaining 20 virulence genes had a positive rate of less than 20% (Table 3).

**TABLE 3:** Virulence genes of 17 mcr-1-positive Enterobacteriaceae.

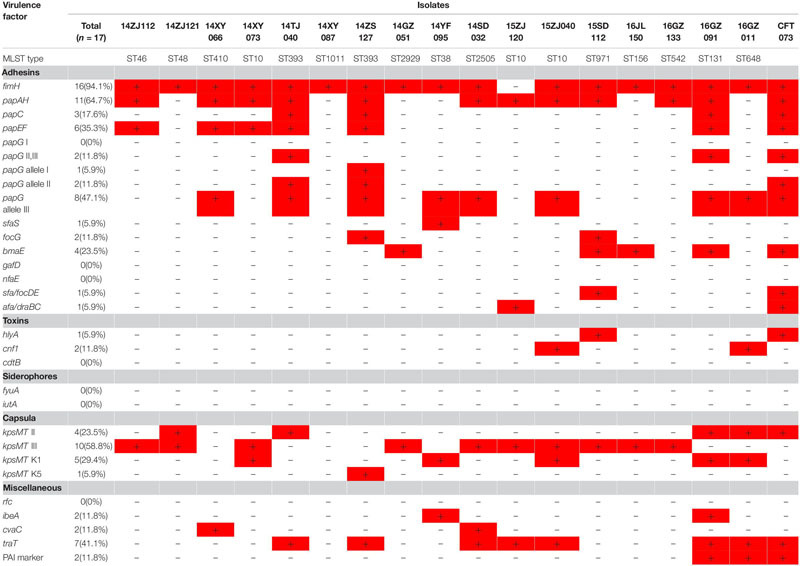

*CFT073 used as VFs control in this study. The Color values are to highlight the positive results of virulence genes.*

## Discussion

The *mcr-1* gene has been mainly detected in *Enterobacteriaceae* including *E. coli*, *K. pneumoniae*, *E. cloacae*, *E. aerogenes*, and *Salmonella enterica* (Gao et al., 2016). *E. coli* is the predominant species among MPE (Chen et al., 2017). At present, several countries have detected the *mcr-1* gene in *Enterobacteriaceae*, mainly in food, animals, healthy people, and the environment (Hasman et al., 2015; Fernandes et al., 2016; Kieffer et al., 2017). However, the isolation rate is low in hospitalized patients (0.1–1.0%) (Prim et al., 2016; He et al., 2017; Quan et al., 2017; Wang et al., 2017; Lu et al., 2018). This study investigated the prevalence of the *mcr-1* gene in patients with intra-abdominal, urinary tract, and respiratory infections in China from 19 hospitals. Our findings point to a low prevalence (17/6401, 0.27%) of *mcr-1*-positive isolates among *Enterobacteriaceae* strains. IAI, UTI, and LRTI were the common infections in the hospital. Our results might reveal a low prevalence of infections caused by MPE in China.

Multi-locus sequence typing analysis showed that 17 MPE isolates belonged to 14 different types, and PFGE and serotyping by O and H antigens both showed high diversity. Among the three ST10 strains, two were isolated from different departments of the same hospital in Zhejiang province 4 months apart (15ZJ120 and 15ZJ040), the genomes of which were of 1376 SNPs. In addition, their PFGE patterns were also of obvious difference; therefore, they were not regarded to be clonal. The two ST393 strains, 14ZS127 and 14TJ040, were from different hospitals. One MPE strain isolated from a urinary tract infection belonged to ST131 which is one of the most important types of extraintestinal infection and has long been recognized as a global threat to human health, often associated with human urinary tract and blood infections (Hasman et al., 2015; Mathers et al., 2015; Shen et al., 2018).

Although most of the MPE strains described to date are resistant to colistin, the present isolates exhibited low-level colistin resistance (MIC: 2–8 μg/ml), and three of them were sensitive to colistin. This is consistent with previous studies showing that *mcr-1*-carrying *E. coli* are not always resistant to colistin (Quan et al., 2017; Li B. et al., 2018). This might be caused by the different *mcr-1* expression level in these isolates. Among these phylogenetically diverse strains, we identified IncX4 (8 strains) and IncI2 (four strains) types of *mcr-1*-carrying plasmids with high similarities, respectively, which were the prevalent *mcr-1*-carrying plasmids (Li et al., 2017; Matamoros et al., 2017; Quan et al., 2017). These indicated that these strains might obtain the plasmids by horizontal gene transfer recently. Therefore, the costs of plasmid fitness might cause different expression levels of the genes in the plasmid (Carroll and Wong, 2018; San Millan and Maclean, 2019), as well as *mcr-1*, leading to the varied colistin resistance although the strains carried the similar *mcr-1*-harboring plasmids.

We found that MPE isolates carried other antimicrobial resistance genes, especially ESBLs, ampC, and carbapenemase genes; particularly in the IncHI2A-type *mcr-1*-harboring plasmids, multiple resistance genes were identified. This was in agreement with previous studies (Mediavilla et al., 2016; Bi et al., 2017; Rhouma and Letellier, 2017; Li Q. et al., 2018; Zheng et al., 2019). A study in China investigated the coexistence of ESBLs and *mcr-1* genes in *E. coli* isolated from chickens between 2008 and 2014 and concluded that *mcr-1*-positive strains carry at least one ESBL (Wu et al., 2018). The 16 MPE isolates in the present study carried at least one ESBL resistance gene or carbapenemase gene, and one isolate only carried *mcr-1*. The most commonly encountered ESBL gene in the present study was *bla*_*TEM*_ (16/17, 94.1%). The coexistence of the *mcr-1* gene with other drug resistance genes increases the possibility of the emergence of pan-drug-resistant bacteria, leading to less choice of therapeutic drugs.

The 17 MPE isolates carried a variety of virulence genes, mainly adhesion-associated and capsular-associated virulence factors. The adhesion VF genes were mainly *fimH* (16/17, 94.1%), followed by *papA* (11/17, 64.7%), *papG* allele III (8/17, 47.7%), and *papEF* (6/17, 35.3%), while the capsular-associated virulence genes were mainly *kpsMT* III (10/17, 58.8%), followed by *kpsMT* K1 (5/17, 29.4%), and *kpsMT* II (4/17, 23.5%). Uropathogenic *Escherichia coli* (UPEC) is the main cause of urinary tract infection, and its virulence factors include adhesion factor, cytotoxic substances, iron carrier, capsule, lipopolysaccharides (Jacobsen et al., 2008). These virulence factors help bacteria break through the host’s immune defense system, colonize the urinary tract, and even invade the epithelial cells of the urethra, leading to urinary tract infection. The adhesion factor is considered to be the most important virulence factor of UPEC. Under the mediation of adhesion factors, UPEC can invade host cells and continue to reproduce to form intracellular colonies, thus avoiding the attack of the host immune system (Lüthje and Brauner, 2014; Subashchandrabose and Mobley, 2015). The main adhesion factors of UPEC are fimbriae (FimABCDEFGH) and pyelonephritis-associated pili (pap). Type I fimbriae mostly in urinary tract infection plays an important role. P pilus mainly plays an important role in upper urinary tract infection and onset of acute pyelonephritis (Snyder et al., 2005; Jacobsen et al., 2008; Lüthje and Brauner, 2014; Subashchandrabose and Mobley, 2015). In the present study, the 17 MPE isolates carried a variety of virulence genes, mainly the pili gene *fimH* (94.1%) and the P-pilin gene *papA* (64.7%), followed by the capsular-related gene *kpsMT* III (58.8%).

## Conclusion

Our findings indicate that the prevalence of MPE in IAI, UTI, and LRTI patients was low in China. However, the sequence profile and mobility analyses of the *mcr-1*-carrying plasmids revealed a frequently horizontal transfer of the plasmids, like the IncX4 and IncI2 plasmids observed in this study, as well as the IncHI2A type MDR plasmids carrying a number of resistance genes. The characteristics of these strains, including high transferability of different resistance genes and multiple virulence factors, can contribute to the rapid spread and increasing mortality of these strains. Controlling of *mcr-1*-positive stains should be an urgent issue.

## Data Availability Statement

All datasets presented in this study are included in the article/Supplementary Material.

## Ethics Statement

The studies involving human participants were reviewed and approved by the Human Research Ethics Committee of Peking Union Medical College Hospital (Number: S-K238). Written informed consent for participation was not required for this study in accordance with the national legislation and the institutional requirements.

## Author Contributions

QY, LX, and YX conceived and designed the work. BJ, QY, PJ, EL, HZ, and DL performed the survey. BJ, PD, and TK analyzed the data and wrote the manuscript. All authors read and approved the final manuscript.

## Conflict of Interest

The authors declare that the research was conducted in the absence of any commercial or financial relationships that could be construed as a potential conflict of interest.

## Footnotes

1 http://enterobase.warwick.ac.uk/species/ecoli/allele_st_search

2 https://cge.cbs.dtu.dk/services/SerotypeFinder/

3 http://blast.ncbi.nlm.nih.gov
