# Supplementary figures and images for: Antimicrobial Susceptibility and Virulence of mcr-1-Positive Enterobacteriaceae in China, a Multicenter Longitudinal Epidemiological Study
Source: Front Microbiol. 2020 Jul 28;11:1611. doi: 10.3389/fmicb.2020.01611 (PMC7399235; doi:10.3389/fmicb.2020.01611)

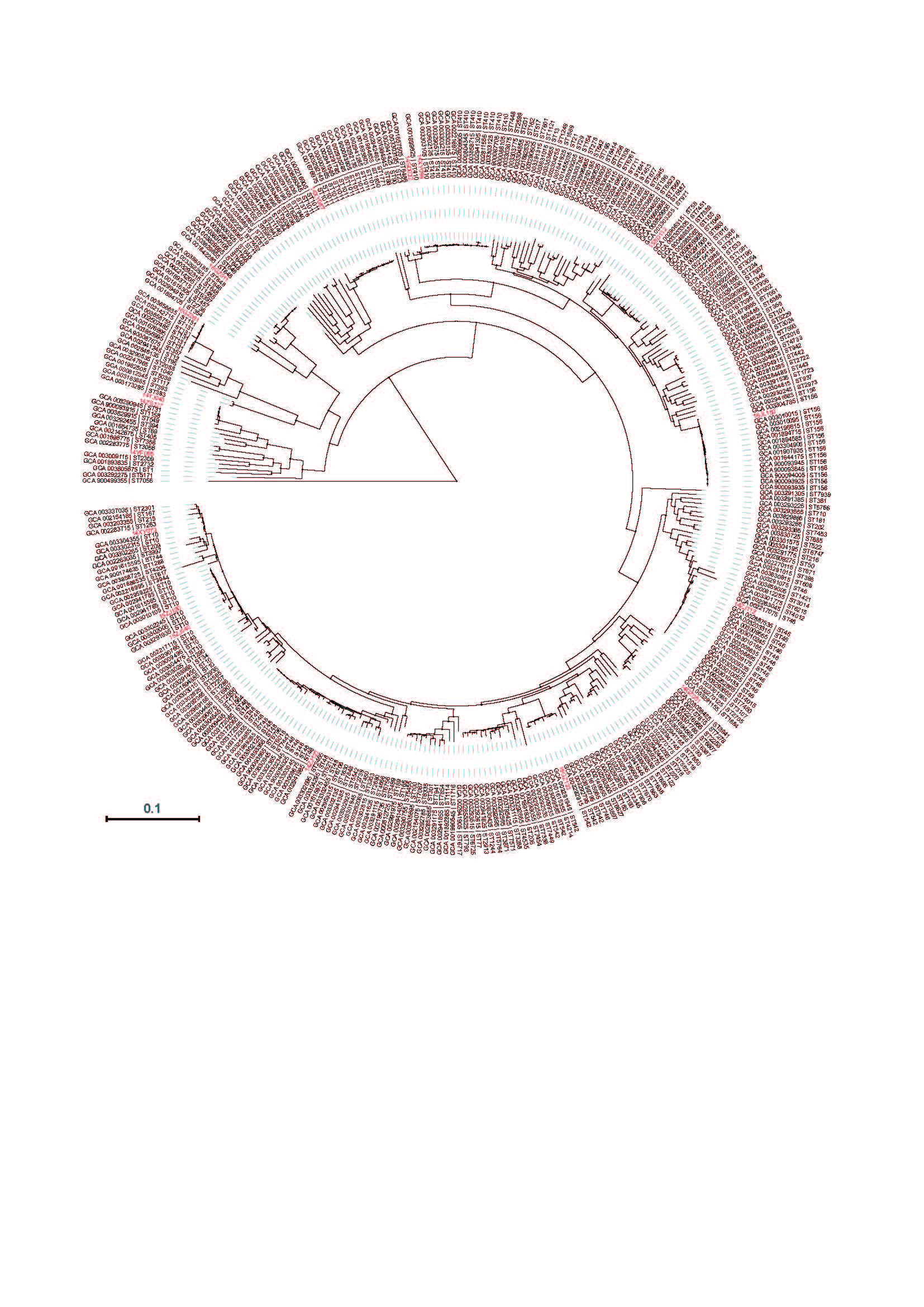

Supplement: Supplementary file 1 [file Image_1.JPEG]
